# Differential Effects of Electroconvulsive Therapy on Patients with Schizophrenia Versus Depressive Disorder: Clinical Distinction Between Antipsychotic and Antidepressant Effects of Electroconvulsive Therapy

**DOI:** 10.3390/brainsci15020126

**Published:** 2025-01-27

**Authors:** Naho Nakayama, Tatsuo Nakahara, Hideyuki Iwanaga, Manabu Hashimoto, Takako Mitsudo, Yoshiomi Imamura, Hiroko Kunitake, Yoshito Mizoguchi, Takefumi Ueno

**Affiliations:** 1 Department of Psychiatry, National Hospital Organization, Hizen Psychiatric Medical Center, 160 Mitsu, Yoshinogari, Kanzaki 842-0192, Saga, Japan; naho.nakayama.h259@gmail.com (N.N.); nakahara@ruby.plala.or.jp (T.N.); hideiwan@gmail.com (H.I.); ma9hashi@yahoo.co.jp (M.H.); mitsudo.takako.692@m.kyushu-u.ac.jp (T.M.); 2 Department of Neuropsychiatry, Graduate School of Medical Sciences, Kyushu University, 3-1-1 Maidashi, Higashi-ku, Fukuoka 812-8582, Japan; 3 Department of Psychiatry, Faculty of Medicine, Saga University, 5-1-1 Nabeshima, Saga 849-8501, Japan; e7730@edu.cc.saga-u.ac.jp (Y.I.); kunihiro.0720@gmail.com (H.K.); ymizo@cc.saga-u.ac.jp (Y.M.)

**Keywords:** brief psychiatric rating scale, electroconvulsive therapy, schizophrenia, major depressive disorder, bipolar disorder, plasma brain-derived neurotrophic factor

## Abstract

Objective: Electroconvulsive therapy (ECT) is utilized for treating psychiatric disorders, such as schizophrenia (SCZ), major depressive disorder (MDD), and bipolar disorder (BD). We aimed to compare pre- and post-ECT treatment outcomes between patients with SCZ and a combined group of patients with MDD and BD (MDD+BD) to assess the distinction between the antipsychotic and antidepressant effects of ECT. Methods: ECT was administered to patients with SCZ (n = 17) and those with MDD+BD (n = 7). Symptoms were evaluated using the brief psychiatric rating scale (BPRS), clinical global impression scale (CGI), and global assessment of functioning (GAF). Plasma brain-derived neurotrophic factor (BDNF) levels were also measured. Results: The BPRS, CGI, and GAF scores significantly differed after ECT compared with those before ECT in each patient group. However, no significant differences were observed between the groups for each disorder. No significant differences were observed in plasma BDNF levels between the groups at baseline and during ECT. At baseline, only depression scores were more favorable in the SCZ group, whereas positive symptoms and disorganization scores were higher in the MDD+BD group. During treatment, positive symptoms, activation, and disorganization items were significantly more favorable in the MDD+BD group compared with the SCZ group. Total BPRS scores were not associated with plasma BDNF levels; however, rating scores of the several items related to activation, resistance, and disorganization were positively correlated with BDNF levels. Conclusion: ECT effects on several clinical outcomes in the MDD+BD group were associated with plasma BDNF levels. These findings suggest that ECT may be more effective for treating MDD than SCZ.

## 1. Introduction

Electroconvulsive therapy (ECT) is widely utilized to treat various psychiatric disorders, including major depressive disorder (MDD) [1,2,3,4], bipolar disorder (BD) [5,6], and schizophrenia (SCZ) [7,8,9]. Recent longitudinal magnetic resonance imaging studies have demonstrated ECT-induced changes in gray matter volume in patients with MDD [10,11,12] and SCZ [13,14,15]. Furthermore, diagnosis-specific differences in volume changes [16] and large-scale network interactions [17] between MDD and SCZ have been elucidated, although the mechanisms underlying the antidepressant and antipsychotic effects of ECT remain incompletely understood. We aimed to determine whether diagnosis-specific brain changes in response to ECT are associated with clinical improvement. However, no direct comparison of ECT responses between SCZ and MDD has been performed using the same rating scales and measuring plasma BDNF levels.

BDNF is a potential biomarker of the effects and clinical response of ECT [18]. BDNF induces a sustained elevation of intracellular Ca^2+^ [19] and might exhibit an anti-inflammatory effect by inhibiting microglial activation [20,21]. These findings suggest that transdiagnostic or diagnosis-specific changes in BDNF could be associated with the clinical effects of ECT. Serum BDNF levels increase with long-term aerobic exercise, possibly because of neural activity associated with physical exercise, which promotes BDNF expression.

In this study, we aimed to compare the clinical efficacy and acceptability of ECT between patients with SCZ and a combined group of patients with MDD and BD. Symptom severity and changes in symptom status were evaluated using the brief psychiatric rating scale (BPRS), which is useful for evaluating patients with MDD, BD, and SCZ [22,23]. We also investigated the relationship between BDNF levels and clinical outcomes before and after ECT. Our study revealed that ECT effects on several clinical outcomes of MDD+BD were associated with plasma BDNF levels. These findings suggest that ECT may be more effective in patients with MDD than in those with SCZ.

## 2. Materials and Methods

All patients included in this study underwent ECT at the Hizen Psychiatric Center, Saga, Japan, between November 2017 and January 2020. ECT was administered to patients who exhibited insufficient response to drug treatment, experienced severe psychiatric symptoms with communication difficulties, or had side effects from drugs. All patients, except for Patient No. 7, underwent ECT for the first time. Written informed consent for participation in the study was obtained from all patients and their relatives. The ethics committee of the hospital approved the study protocol.

Of the 27 participants, 18 were diagnosed with SCZ or schizoaffective disorder, whereas 8 and 1 with somatic symptom disorder were diagnosed with MDD or BD, respectively, based on the Diagnostic and Statistical Manual of Mental Disorders, Fifth Edition (DSM-5) [24]. The patient profiles were categorized into three groups: 17 with SCZ, 7 with MDD+BD, and 3 in the excluded group. The overview included information on age, sex, disease duration, number of ECT sessions, prescribed medications, and side effects. Table 1 outlines the reasons for excluding patients. The results were compared between the SCZ and MDD+BD groups. The MDD+BD group had a significantly higher age and age at onset, whereas the number of hospitalizations was significantly lower. The frequency of ECT varied based on the patients’ clinical status, but no significant differences were observed in the number of ECT sessions between the two groups (Table 2).

Experienced clinicians used the BPRS, clinical global impression scale (CGI), and global assessment of functioning (GAF) to assess symptoms and disease severity. A 6-factor model of the 24-item BPRS (effect, positive symptoms, negative symptoms, activation, resistance, and disorganization) was utilized [25].

ECT was performed using bilateral electrodes (pulse width: 0.50 ms) or right unilateral electrodes (pulse width: 0.25 ms). Remifentanil (1 g/kg/min div) and propofol (0.5 kg) were administered intravenously as anesthesia, whereas rocuronium (0.6 kg) was administered intravenously as a muscle relaxant. Three out of seventeen patients in the SCZ group, two out of seven patients in the MDD+BD group, and one out of three in the exclusion group underwent right-sided electrode ECT.

Blood samples for BDNF measurement were collected 0–14 days before ECT and 3–14 days after ECT. Venous blood was collected in blood collection tubes containing EDTA 2Na and aprotinin, then centrifuged at 3500× *g* for 15 min at 4 °C, and the supernatant was used for plasma analysis. Plasma BDNF concentrations were measured using a commercially available enzyme-linked immunosorbent assay (ELISA) kit (Mature BDNF ELISA Kit Wako 296-83201, Fujifilm Wako Pure Chemical Industries, Ltd., Osaka, Japan).

Briefly, standards and samples were added to the 96-well plates, incubated, and shaken for 2 h at room temperature. After washing with wash buffer, the plates were incubated for 2 h with Anti-Human BDNF polyclonal antibody. Subsequently, the plates were incubated for 1 h with Streptavidin–HRP conjugate working solution, and TMB substrate solution was added to the well to develop color. The reaction was stopped with 0.25 N HCl, and the absorbance was read at 450 nm on a microplate reader (Multiskan FC, Thermo Fisher Scientific, Tokyo, Japan). BDNF concentrations were determined automatically according to the BDNF standard curve (ranging from 23.4 pg to 1500 pg purified BDNF).

All samples were analyzed in duplicate in one session. The average values of plasma BDNF for pre-ECT (SCZ: 682 ± 527 pg/mL and MDD+BD: 484 ± 228 pg/mL) were close to the average value (747 ± 193 pg/mL) and within the range (170–1126 pg/mL) previously reported [26]. All samples were anonymized, and no clinical parameters or patient data were available.

JMP18.1.1 software (SAS Institute, Cary, NC, USA) was utilized for all statistical analyses. Analysis of variance (ANOVA) was used to compare BPRS, CGI, GAF, and BDNF between the groups, and the mean, standard deviation, F-value, and *p*-value were obtained. Cohen’s d and 95% confidence intervals (CIs) used in the forest plot were calculated from the sample size, mean, and standard deviation of each group [27]. Regression analysis was used to determine the correlation between BDNF blood concentration and BPRS sub-scores, yielding the correlation coefficient and *p*-value.

G*Power 3.1.9.6 software for Mac (Heinrich-Heine-Universität Düsseldorf, Düsseldorf, Germany) (https://www.psychologie.hhu.de/arbeitsgruppen/allgemeine-psychologie-und-arbeitspsychologie/gpower, accessed on 15 January 2025) was used to conduct a post hoc analysis to compute the achieved power (1-β error probability) with an α error probability of 0.05 and an effect size (f) calculated from the number of groups, means, and sample sizes for the ANOVA of BPRS, CGI, and CAF, as well as linear correlations between BDNF levels and BPRS sub-scores.

## 3. Results

**Figure 1:**
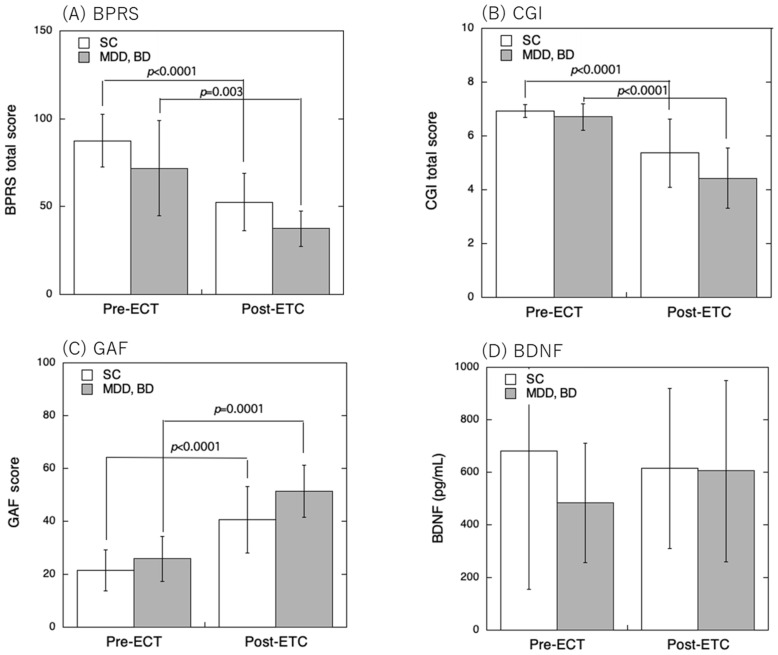
Effects of ECT on BPRS (**A**), CGI (**B**), and GAF (**C**) scores as well as plasma BDNF levels (**D**) of patients with schizophrenia (SCZ) and those with depressive (MDD) and bipolar (BD) disorder.

No significant differences were observed in the rating scales before ECT compared with those after ECT for each patient group; however, no significant differences were observed between the groups for each disorder (Figure 1). Additionally, no significant differences were observed between the SCZ and MDD+BD groups in pre-ECT BPRS (*p* = 0.195), CGI (*p* = 0.938), GAF (*p* = 0.758), and BDNF (*p* = 0.736). Similarly, no significant differences were observed in post-ECT BPRS (*p* = 0.222), CGI (*p* = 0.116), GAF (*p* = 0.103), and BDNF (*p* = 1.000).

No significant differences in plasma BDNF levels were observed between the groups at baseline, and no significant variation occurred in any group during treatment (Figure 1). The power for the ANOVA of BPRS, CGI, and CAF was 0.999.

However, the power for BDNF was 0.119, indicating that a sample size of 592 participants would be required to achieve the desired statistical power. A recent meta-analysis examining the correlation between ECT treatment and BDNF levels has revealed that a significant increase in BDNF levels after ECT was reported in only two studies. In contrast, four other studies revealed slight changes in BDNF levels, with no statistical significance [28].

**Figure 2:**
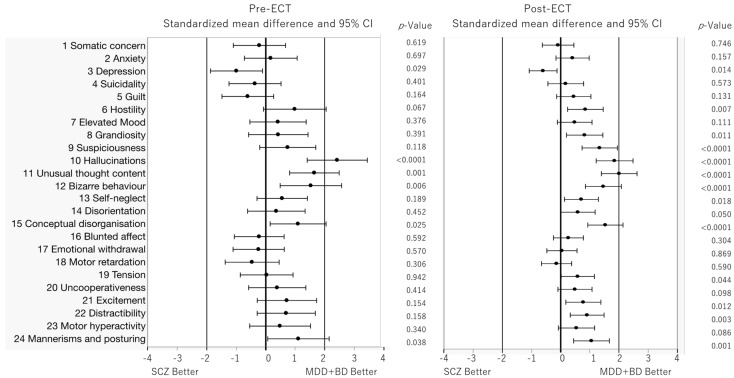
Forrest plot comparison of pre- and post-ECT. Standardized mean differences in BPRS sub-scores (Cohen’s d) between schizophrenia (SCZ) and depressive disorder and bipolar disorder (MDD, BD) are plotted.

We used Cohen’s d effect size to reanalyze the 24 individual BPRS items and estimate the difference between the SCZ and MDD+BD groups. This analysis involved subtracting the rating scales for patients with MDD and BD from those for patients with SCZ. Thus, negative values represent the favoring of psychopathology in the schizophrenia group, and vice versa (Figure 2). At baseline, only depression scores showed negative values (*p* = 0.029), favoring the SCZ group, whereas hallucinations (*p* < 0.0001), unusual thought content (*p* = 0.001), bizarre behavior (*p* = 0.006), conceptual disorganization (*p* = 0.025), and mannerisms and posturing (*p* = 0.038) showed positive values, favoring the MDD+BD group. During treatment, several additional items beyond those identified at baseline showed significant differences between the groups, with positive values favoring the MDD+BD group. Compared with the SCZ group, the MDD+ BD group exhibited higher scores for hostility (*p* = 0.007), grandiosity (*p* = 0.011), suspiciousness (*p* < 0.0001), hallucinations (*p* < 0.0001), unusual thought content (*p* < 0.0001), bizarre behavior (*p* < 0.0001), self-neglect (*p* = 0.018), disorientation (*p* = 0.050), conceptual disorganization (*p* < 0.0001), tension (*p* = 0.044), excitement (*p* = 0.012), distractibility (*p* = 0.003), and mannerisms and posturing (*p* = 0.001). Only depression showed negative values (*p* = 0.014), favoring SCZ at post-ECT.

A tendency for increased BPRS levels in patients with MDD+BD was observed after ECT, although the difference was insignificant. Therefore, correlations between BDNF levels and BPRS sub-scores were estimated, yielding the following results (Table 3): Rating scales of hostility (*p* = 0.0006), suspiciousness (*p* = 0.0260), conceptual disorganization (*p* = 0.0409), tension (*p* = 0.0228), uncooperativeness (*p* = 0.0006), excitement (*p* < 0.0001), and mannerisms and posturing (*p* = 0.0139) were positively correlated with BDNF levels in MDD+BD patients after ECT. Only suspiciousness sub-score (*p* = 0.0154) was correlated with BDNF levels in patients with SCZ. These findings suggest that BDNF may be involved in the effects of ECT in patients with MDD and BD, but not in patients with SCZ. The powers for the linear correlations between BDNF and BPRS sub-scores were as follows: For all items in the SCZ (n = 17) and MDD+BD (n = 7) groups at pre-ECT, the power was less than 0.5. The power for suspiciousness in the SCZ (n = 45) group at post-ECT was 0.716. For hostility, it was 0.979; for suspiciousness, 0.605; for conceptual disorganization, 0.518; for tension, 0.630; for uncooperativeness, 0.979; for excitement, 0.999; and for mannerisms and posturing, 0.716 for the MDD+BD (n = 20) group at post-ECT.

## 4. Discussion

ECT was effective in treating patients with SCZ; however, the SCZ group exhibited significantly lower responses to ECT regarding positive symptoms, activation, and disorganization, except for depressive behavior, compared to the MDD+BD group. Gray matter volume (GMV) has been reported to increase after ECT in the MDD and SCZ groups, indicating that some mechanisms of ECT are common across diagnoses, whereas others differ between diagnoses. Specifically, alterations in GMV within the left pregenual anterior cingulate cortex were identified as unique to SCZ and were significantly correlated with percentage changes in BPRS scores [16]. In MDD, ECT-induced changes include an increased somatomotor network (SMN) to visual network (VIN) connection, increased self-connection within the default mode network (DMN), and decreased connectivity from the limbic network (LIN) to multiple networks and from the frontoparietal network to the SMN. Additionally, decreased self-connection within the dorsal attention network and the LIN was observed in MDD compared with SCZ. Self-connection within the dorsal attention network was positively associated with scores on the Beck Depression Inventory (revised version). Conversely, self-connection within the DMN showed a positive association, and self-connection within the LIN exhibited a negative association with the positive and negative syndrome scale positive symptom scores in individuals with SCZ. Furthermore, the SMN-to-VIN connection demonstrated a positive association, whereas the VIN-to-DMN connection and self-connection within the DMN showed negative associations with the positive and negative syndrome scale general psychopathology scores in individuals with SCZ [17].

The diagnosis-specific effects of ECT observed in this study might be related to GMV changes or aberrant large-scale network interactions, although a direct explanation remains impossible. Thus, comparing post-ECT changes between the two diagnostic groups provides insights into diagnosis-specific changes and transdiagnostic changes.

Compared with healthy controls, patients with MDD and BD have been reported to share common differences in nodal parameters within the right amygdala and right parahippocampal gyrus, suggesting that patients with MDD and BD can be considered a combined group for certain analyses [29].

BDNF induces long-lasting Ca^2+^-activated K^+^ currents and sustained elevation of intracellular Ca^2+^ [19]. BDNF also impacts intracellular Ca^2+^ signaling in microglial cells [20,21], which may be important in regulating inflammatory responses and may contribute to the pathophysiology and treatment of psychiatric disorders. Additionally, BDNF induces a rapid increase in the total number of cell surface GABA(A) receptors by activating Trk B receptors [30], suggesting a relationship between GABA(A) receptor deficits and central nervous system disorders [31]. The pathophysiology of SCZ is related to inflammatory responses mediated by microglia and intracellular Ca^2+^ signaling [32]. However, our results showed no significant change in BDNF levels in SCZ patients before and after ECT. Conversely, no association of pro BDNF and BDNF levels with depressive state was reported [33], although the present results elucidated positive correlations between BDNF levels and BPRS sub-scores in patients with MDD+BD after ECT. Our results might indicate a diagnosis-specific change in BDNF after ECT. The ECT-induced change in BDNF levels has been reported in patients with MDD [34] and SCZ [35], though the results were conflicting [36,37].

A 2019 Cochrane review revealed that ECT has a positive effect on medium-term clinical response for patients with treatment-resistant schizophrenia (TRS); however, clear and convincing advantages or disadvantages of adding ECT to standard care for other outcomes are lacking [7]. Conversely, ECT has been proven effective in patients with treatment-resistant depression (TRD) [1,38]. Searches of the PubMed database were conducted in June 2023 using the medical subject headings terms “ECT”, “treatment-resistant depression (TRD)”, and “treatment-resistant schizophrenia (TRS)”. The search identified 7994 TRD reports, including 761 ECT reports, and 2857 TRS reports, including 202 ECT reports. Therefore, the percentage of “ECT” in “TRD” was larger than that in “TRS” (9.9% vs. 7.1%). These identified publications suggest that the utilization rate of ECT may be lower for SCZ treatment than for MDD+BD treatment.

The utilization and practice of ECT worldwide indicate that affective disorders (unipolar/bipolar depression) are the main diagnoses in Australia, New Zealand, the United States of America, and Europe, whereas SCZ is the main diagnosis in Asia overall [39]. In Western countries, most patients with MDD are older women, whereas in Asian countries, younger men with schizophrenia constitute the majority [4].

Predicting the therapeutic efficacy of ECT using brain imaging after ECT will be feasible when the brain sites associated with antipsychotic and antidepressant effects are identified. This finding might provide an effective means of treating treatment-resistant depression and treatment-resistant SCZ, including cases requiring clozapine.

## 5. Limitations

We grouped depression and bipolar affective disorder together. The breakdown of cases is as follows: depression (F32.9), three cases; bipolar affective disorder (F31.9), two cases; bipolar affective disorder with severe depressive episodes without psychotic symptoms (F31.4), one case; and bipolar affective disorder with severe depressive episodes with psychotic symptoms (F31.5), one case. In general, manic episodes last from 2 weeks to 4 to 5 months, whereas depressive episodes typically last approximately 6 months. Particularly in the elderly, depressive episodes can last for more than a year. The ages of the F31.9 cases are 57 and 63, which are considered elderly, suggesting that their depressive states may continue for a relatively long time. As F31.4 represents severe depression without psychotic symptoms, these three cases can be included in the depression group. The issue is with F31.5, but we have included this case in the group. The F32.3 case exhibited severe psychiatric symptoms and was treated with risperidone, so it was added to the SCZ group. Also, there are results that show that ECT is more effective in older groups for depression [40], so it is possible that the MDD+BD group may have a higher effect than the SCZ group. These points should be noted to ensure careful interpretation of the results.

The SCZ group primarily included patients with SCZ plus schizoaffective disorder. It is more appropriate to divide this group into patients with affective symptoms and those with psychotic symptoms.

In this study, clinical evaluations of the SCZ and MDD groups were performed using the BPRS. To enhance the reliability and depth of ECT effect assessments, additional validation with scales such as the Hamilton depression rating scale, the positive and negative syndrome scale, and the negative syndrome scale is necessary. A single healthcare staff member performed all clinical evaluations, and inter-rater reliability tests were not conducted. Concerns regarding the consistency and reproducibility of the clinical assessments should be considered.

## 6. Conclusions

ECT was effective for patients with SCZ and MDD+BD; however, the positive symptoms, activation, and disorganization responses to ECT were significantly higher in the MDD+BD group than in the SCZ group, except for depressive behavior. Additionally, the ECT effects for several clinical outcomes of MDD+BD were associated with plasma BDNF levels. These findings suggest that ECT may be more effective in patients with MDD than in those with SCZ. The implications of these findings should be discussed in the broadest context possible. Future research directions may also be highlighted.

## Figures and Tables

**Table 1:** Participant demographic characteristics.

Patients	No	Age	Sex	Diagnosis	Diagnosis (F-Code)	Onset Age (y)	Illness Period Until ECT (y)	Number of Hospitalizations	Electrode Arrangement	Number of Acute ECT Treatments	Number of Continuation ECT Treatments	Number of Maintenance ECT Treatments	Concomitant Psychotropics		Disorganization Responses to ECT
													before	after	
1	1	27	M	SCZ	SCZ (F20.0)	20	7	2	bf	12	-	-	HPD 9 mg, Que 400 mg	HPD 3 mg, Ola 20 mg	-
2	3	26	M	SCZ	SCZ (F20.9)	18	8	2	bf	15	18	10	Ola 20 mg, HPD 9 mg	Ola 20 mg, Lam 200 mg, LPZ 50 mg	-
3	4	64	M	SCZ	SCZ (F20.2)	28	36	5	bf	11	8	-	Zot 75 mg	Zot 75 mg	-
4	5	32	M	SCZ	SCZ (F20.9)	25	7	2	bf	12	25	2	Ris 12 mg, Que 200 mg	Ris 10 mg, HPD 4.5 mg	-
5	7	19	M	SCZ	SCZ (F20.9)	15	7	4	bf	12	-	-	Clo 600 mg	Clo 550 mg	
6	8	30	M	SCZ	SCZ (F20.9)	24	6	6	bf	27	10	-	Zot 150 mg, HPDinj 5 mg	Ola 20 mg, Zot 150 mg, HPDinj 5 mg	Frequent epileptic wave, cognitive function decline
7	11	44	M	SCZ	SCZ (F20.9)	16	28	17	bf	12	-	-	Ris 12 mg, Ase 20 mg	Ris 12 mg, Ase 20 mg	First ECT started in 2011
8	12	45	F	SCZ	SCZ (F20.9)	22	23	45	bf	19	17	-	Ola 5 mg, Ris 1 mg	Ola 20 mg,	
9	23	45	M	SCZ	SCZ (F20.9)	18	17	18	bf	28	-	-	Clo 400 mg, Lam 300 mg	Clo 400 mg, Lam 300 mg	Memory impairment present
10	26	64	M	SCZ	SCZ (F20.2)	30	34	2	ru	12	5	4	Ase 15 mg, CPZ 100 mg	Ase 15 mg, CPZ 100 mg	
11	28	69	F	SCZ	SCZ (F20.9)	31	38	11	ru	12	-	-	Olz 20 mg	Olz 15 mg	Slowing of brain waves on EEG
12	29	47	F	SCZ	SCZ (F23.9)	47	0	1	bf	15	-	-	Que 300 mg	Que 750 mg, Pal 3 mg	
13	31	50	M	SCZ	SCZ (F20.9)	19	31	13	bf	15	-	-	Clo 500 mg	Clo 500 mg	Slowing of brain waves on EEG
14	9	20	F	SCZ	SCZ (F20.9)	19	1	11	bf	13	13	-	Ola 5 mg	Ola 5 mg, HPDinj 20 mg	Elevated BDNF levels due to hemolysis
15	16	38	F	SCZ	SCZ (F20.9)	23	15	8	bf	16	-	-	CPZ 50 mg, Zot 50 mg	CPZ 150 mg	Slowing of brain waves on EEG Elevated BDNF levels due to hemolysis
16	20	52	F	SCZ	SCZ (F20.9)	19	33	5	bf	23	-	-	Ola 6.25 mg, Ris 2 mg	Ola 20 mg	Elevated BDNF levels due to hemolysis
17	6	61	M	MDD+BD	BD (F31.4)	44	47	6	bf	15	8	6	Mir 30 mg	Lam 100 mg	
18	10	70	F	MDD+BD	MDD (F32.9)	52	18	7	bf	7	-	-	Ola 5 mg, Mir 45 mg	Ola 5 mg, Mir 30 mg	
19	13	57	M	MDD+BD	BD (F31.9)	39	18	2	bf	26	8	-	Mil 50 mg		
20	15	68	F	MDD+BD	MDD (F32.9)	67	1	3	bf	12	5	5	Esc 20 mg, Ris 8 mg, Ven 225 mg	Esc 20 mg, Que 12.5 mg	
21	17	61	F	MDD+BD	MDD (F32.9)	60	1	3	bf	13	8	-	Mir 30 mg, Ola 20 mg	Mir 30 mg, Ola 20 mg	Memory impairment present
22	21	70	M	MDD+BD	BD (F31.5)	69	1	1	ru	15	8	-	Esc 20 mg, Mir 45 mg, Ari 3 mg	-	
23	24	63	F	MDD+BD	BD (F31.9)	50	13	3	ru	10	12	-	VPA 800 mg, Que 112.5 mg	VPA 600 mg, Que 112.5 mg	
24	30	67	F	SCZ	MDD (F32.3)	66	1	1	ru	9	-	-	Ris 1 mg, Esc 20 mg	Esc 20 mg	
Excluded group															
25	2	68	F		BD (F31.9)				bf	7			Ari 3 mg		Discontinued due to liver dysfunction and fever
26	14	57	F		SSD (F45.9)				ru	2			Que 125 mg, Mir 15 mg		Discontinued due to eye pain and discomfort in the mouth
27	19	75	F		SC (F20.9)				bf	2			Ris 2 mg, LPZ 25 mg		Interrupted due to bradycardia and cardiac arrest

Abbreviations: Ari, aripiprazole; Ase, asenapine; BD, bipolar depression; bf, bilateral frontal; Clo, clozapine; CPZ, chlorpromazine; Esc, escitalopram; F, female; HPD, haloperidol; M, male; MDD, major depressive disorder; Lam, lamotrigine; LPZ, levomepromazine; Mil, milnacipran; Mir, mirtazapine; Ola, olanzapine; Pal, paliperidone; Que, quetiapine; Ris, risperidone; ru, right unilateral; SCZ, schizophrenia; SSD, somatic symptom disorder; Ven, venlafaxine; VPA, valproic acid; y, years; Zot, zotepine.

**Table 2:** Comparison of patient profiles between SCZ and MDD+BD groups.

Diagnosis	SCZ	MDD+BD	*p*-Value
Age (y)	43.4 ± 16.2	64.2 ± 5.1	0.0035 *
Sex (M%)	58.8	42.8	
Onset age (y)	25.8 ± 12.8	54.4 ± 11.3	<0.0001 *
Illness period until ECT (y)	17.1 ± 13.7	14.1 ± 16.4	0.6462
Number of hospitalizations	9.0 ± 10.7	3.5 ± 2.1	0.0249 *
Number of acute ECT treatments	15.4 ± 5.6	14.0 ± 6.0	0.5719
Number of continuation ECT treatments	12.0 ± 7.9	8.1 ± 2.2	0.2783
Number of maintenance ECT treatments	5.3 ± 4.1	5.5 ± 0.7	0.9608

* indicates a statistically significant difference between the two groups at *p* < 0.05.

**Table 3:** Correlation coefficient (r) and *p*-value of relationships between BDNF levels and BPRS sub-scores for patients with SCZ and MDD+BD at pre- and post-ECT.

	Pre-ECT				Post-ECT			
BPRS	SCZ		MDD+BD		SCZ		MDD+BD	
Subitem	r	*p*	r	*p*	r	*p*	r	*p*
1 Somatic concern	0.1671	0.5681	−0.2155	0.6816	−0.0500	0.7714	−0.0254	0.9200
2 Anxiety	−0.1109	0.7056	−0.3383	0.5178	0.1040	0.5462	0.1392	0.5818
3 Depression	0.1017	0.7294	−0.4929	0.3205	0.0585	0.7345	0.1130	0.6553
4 Suicidality	0.1245	0.6716	−0.2386	0.6489	0.2286	0.1798	0.3256	0.1873
5 Guilt	0.0373	0.8990	−0.3973	0.4354	0.0501	0.7713	0.0946	0.7087
6 Hostility	−0.4240	0.1308	0.6171	0.1918	0.1231	0.5235	0.7283	0.0006 *
7 Elevated mood	−0.4662	0.0928	0.7732	0.0713	−0.0844	0.6243	−0.1240	0.6236
8 Grandiosity	−0.0822	0.7799	0.6223	0.1870	0.0118	0.9443	−0.1240	0.4593
9 Suspiciousness	−0.0101	0.9728	0.1038	0.8448	0.4007	0.0154 *	0.5229	0.0260 *
10 Hallucinations	0.1377	0.2320	0.2671	0.6088	0.2286	0.1800	0.3256	0.1873
11 Unusual thought content	−0.2588	0.8618	−0.1111	0.8839	0.1473	0.3911	0.2277	0.3635
12 Bizarre behavior	−0.3672	0.1963	−0.0266	0.9599	0.1970	0.2494	0.0737	0.7714
13 Self-neglect	0.2665	0.3571	−0.4150	0.4132	0.0493	0.7752	0.0538	0.8320
14 Disorientation	−0.1670	0.5680	−0.2585	0.6208	0.2666	0.1159	0.4477	0.0624
15 Conceptual disorganization	−0.0634	0.8293	−0.4044	0.4264	0.0839	0.6265	0.4859	0.0409 *
16 Blunted effect	−0.1171	0.6899	−0.4383	0.3846	−0.1109	0.5192	0.4145	0.0872
17 Emotional withdrawal	0.1111	0.7054	−0.7229	0.1045	−0.0510	0.7671	0.3649	0.1365
18 Motor retardation	−0.0316	0.9144	−0.2155	0.6816	0.1787	0.297	0.3470	0.1583
19 Tension	0.3357	0.2406	−0.3310	0.5215	−0.1649	0.3364	0.5327	0.0228 *
20 Uncooperativeness	0.1258	0.6682	0.5852	0.2224	0.1522	0.8062	0.7282	0.0006 *
21 Excitement	−0.3584	0.2082	0.4334	0.3906	0.0292	0.8658	0.8775	<0.0001 *
22 Distractibility	0.0985	0.7374	−0.5905	0.2172	−0.1809	0.2907	0.3066	0.2159
23 Motor hyperactivity	−0.0779	0.7912	0.5700	0.2376	−0.0833	0.6289	0.3256	0.1873
24 Mannerisms and posturing	0.0207	0.9944	0.1541	0.7707	−0.1301	0.4492	0.5680	0.0139 *

* indicates a statistically significant difference between the two groups at *p* < 0.05.

## Data Availability

The data presented in this study are only available on request from the corresponding authors because the data are not publicly available due to privacy or ethical restrictions.

## Abbreviations

BD: bipolar depression; BDNF, brain-derived neurotrophic factor; BPRS, brief psychiatric rating scale; CGI, clinical global impression scale; CPZ, chlorpromazine; DMN, default mode network; ECT, electroconvulsive therapy; GAF, global assessment of functioning; GMV, gray matter volume; MDD, major depressive disorder; LIN, limbic network; SCZ, schizophrenia; SMN, somatomotor network; TRS, treatment-resistant schizophrenia; VIN, visual network.
